# Supplementary material for: A Nasal Brush-based Classifier of Asthma Identified by Machine Learning Analysis of Nasal RNA Sequence Data
Source: Sci Rep. 2018 Jun 11;8:8826. doi: 10.1038/s41598-018-27189-4 (PMC5995932; doi:10.1038/s41598-018-27189-4)
Supplement: Supplementary file 1 — Supplementary Information [file 41598_2018_27189_MOESM1_ESM.pdf]

Supplementary Materials

A Nasal Brush-based Classifier of Asthma Identified by Machine Learning Analysis of Nasal RNA Sequence Data

Authors: Gaurav Pandey[1], Om P. Pandey[1], Angela J. Rogers[2], Mehmet E. Ahsen[1], Gabriel E. Hoffman[1], Benjamin A. Raby[3], Scott T. Weiss[3], Eric E. Schadt[1], Supinda Bunyavanich[1,4]*

*To whom correspondence should be addressed: Supinda Bunyavanich, MD, MPH, Icahn School of Medicine at Mount Sinai, 1425 Madison Avenue #1498, New York, NY 10029, USA, Tel. +1 212 659 8262, Fax +1 212 426 1902, supinda@post.harvard.edu

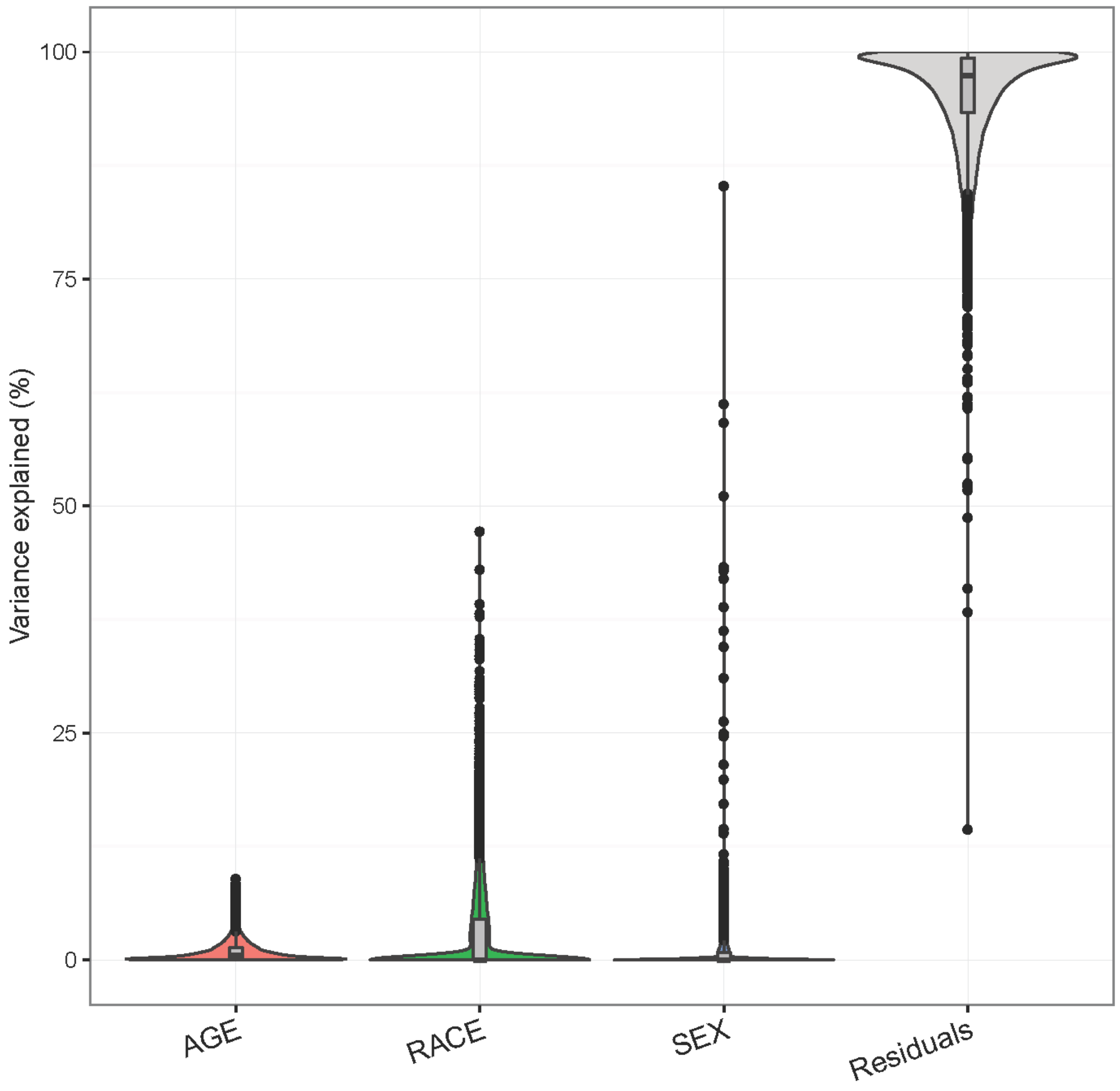

Supplementary Figure 1: variancePartition analysis of the RNAseq development set. Gene expression variation across RNA samples due to age, race, and sex was assessed by variancePartition and found to be minimal.

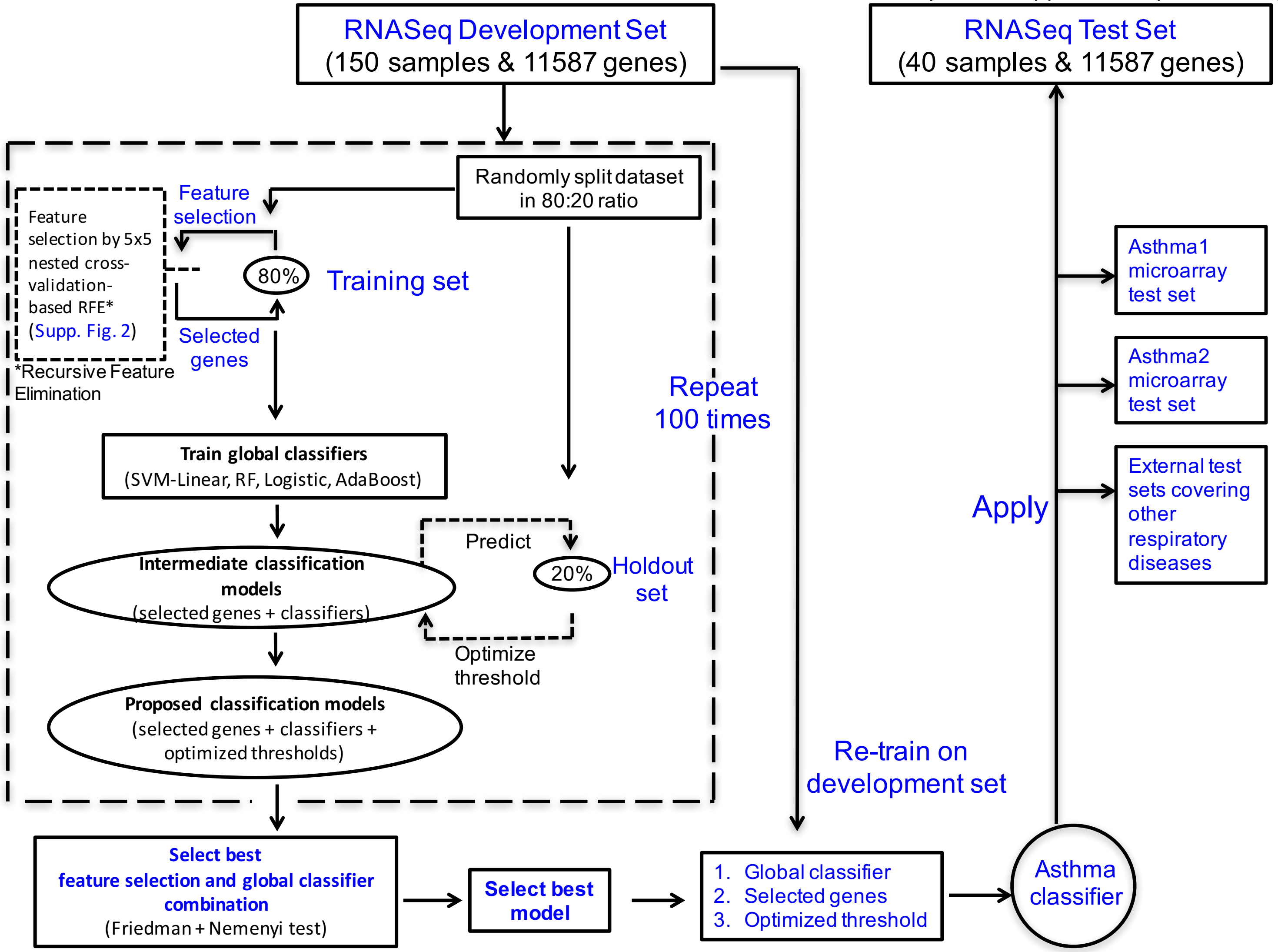

Supplementary Figure 2: **Visual description of the machine learning pipeline used to select predictive features (genes) and develop classification models based on them in the RNAseq development set**. By considering 100 splits of the development set into training and holdout sets (dotted box), many such models were evaluated for classification performance and then compared statistically using Friedman and Nemenyi tests. From this comparison, the best combination of predictive genes and global classification algorithms was determined, which was then executed on the development set to train the final asthma classifier model. This model was applied to an independent RNAseq test set and external microarray-derived cohorts with asthma and other respiratory conditions for final evaluation.

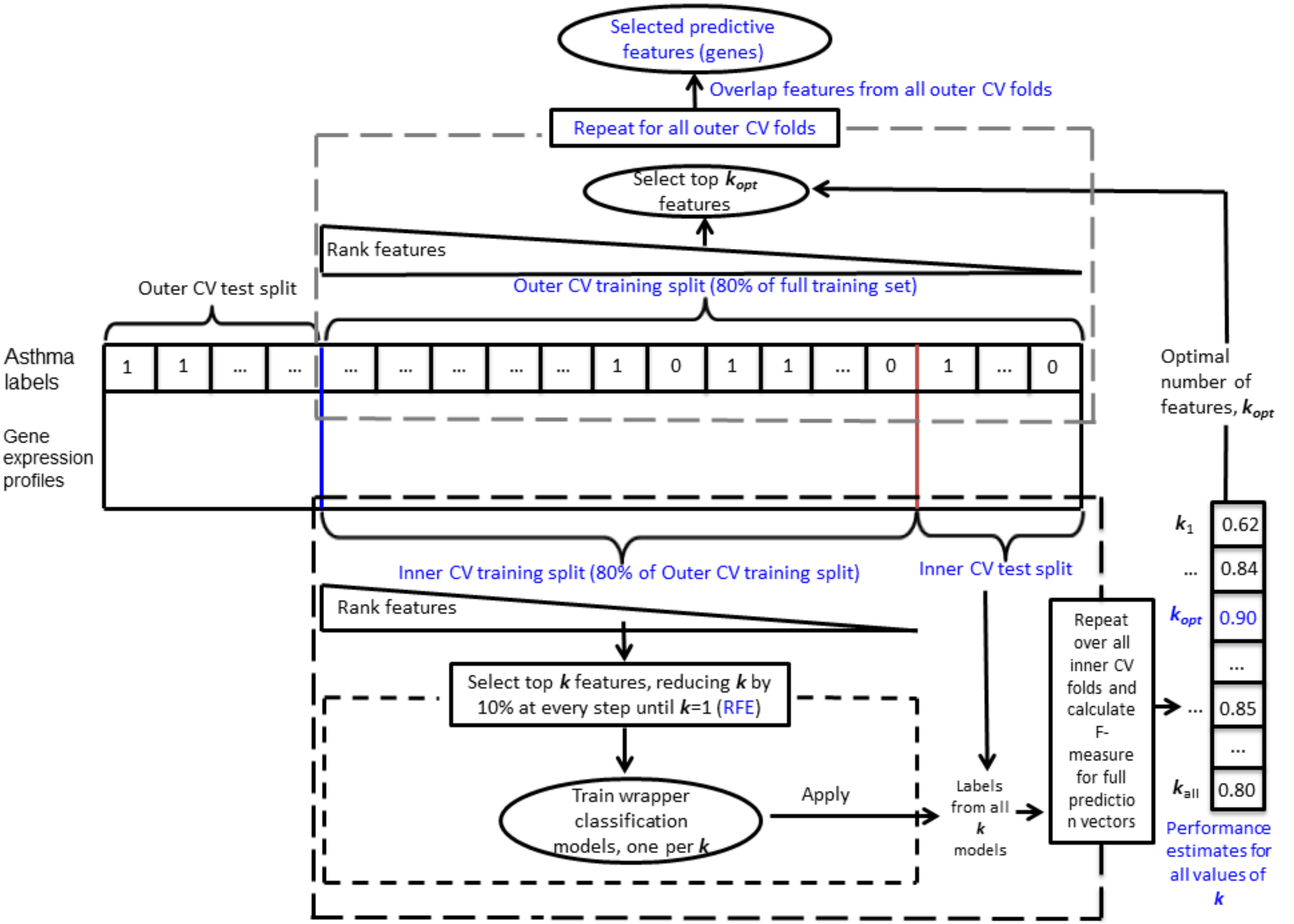

Supplementary Figure 3: **Visual description of the feature (gene) selection component of the machine learning pipeline**. Given a training set, this component used a 5x5 nested (outer and inner) cross-validation (CV) setup to select sets of predictive features (genes). The inner CV round was used to determine the optimal number of features to be selected, and the outer round was used to select the set of predictive genes based on this number, thus reducing the cumulative effect of potential sources of overfitting. The selection of features itself was performed using the Recursive Feature Elimination (RFE) algorithm in combination with wrapper Logistic Regression and SVM with Linear kernel classification algorithms.

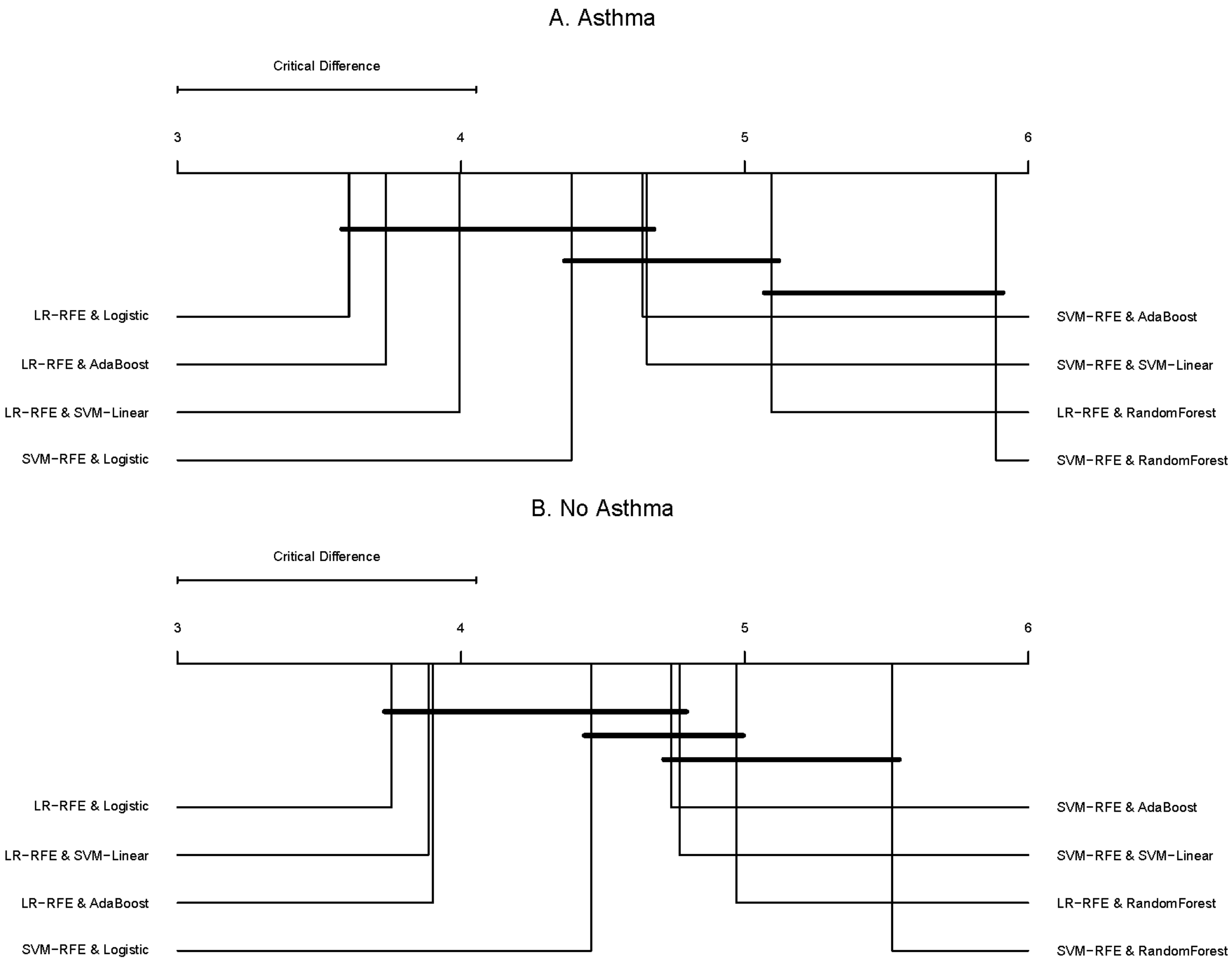

Supplementary Figure 4: **Critical Difference plots demonstrating the statistical comparison of the performance of 100 asthma classification models obtained by various combinations of feature selection and global classification algorithms.** To emphasize the need for parsimony (small feature (gene) sets) in these models, an adapted performance measure defined as the F-measure for each model divided by the number of genes in that model was used for this comparison. The Friedman followed by Nemenyi tests were used to statistically compare these adapted measures and obtain the p-values constituting the above plot. Each combination is represented individually by vertical+horizontal lines on the (A) asthma and (B) no asthma classes constituting the RNAseq development set. Combinations with improving performance are displayed from left to right in terms of the average rank obtained by each of their 100 models, and the combinations connected by thick black lines indicate equivalent statistical performance. The LR-RFE & Logistic model was determined to be the best performing combination, since, on average, it achieved the best performance with the fewest selected genes. LR = Logistic Regression. SVM = Support Vector Machine. RFE = Recursive Feature Elimination.

	PREDICTED CLASS		
ACTUAL CLASS		Class=Yes	Class=No
	Class=Yes	a (True Positive)	b (False Negative)
	Class=No	c (False Positive)	d (True Negative)

$$\text{Precision}_{YES}\ (\text{p}_{YES}) = PPV = \frac{a}{a+c} \qquad \text{Precision}_{NO}\ (\text{p}_{NO}) = NPV = \frac{d}{b+d}$$

$$\text{Recall}_{YES}\ (\text{r}_{YES}) = Sensitivity_{YES} = \frac{a}{a+b} \qquad \text{Recall}_{NO}\ (\text{r}_{NO}) = Sensitivity_{NO} = \frac{d}{c+d}$$

$$\text{F-measure}_{YES}\ (\text{F}_{YES}) = \frac{2r_{YES}p_{YES}}{r_{YES}+p_{YES}} = \frac{2a}{2a+b+c} \qquad \text{F-measure}_{NO}\ (\text{F}_{NO}) = \frac{2r_{NO}p_{NO}}{r_{NO}+p_{NO}} = \frac{2d}{2d+b+c}$$

Supplementary Figure 5: Evaluation measures for classification models. The relationships between F-measure, sensitivity, precision, recall, positive predictive value, and negative predictive value are summarized. F-measure, which is a harmonic (conservative) mean of precision and recall that is computed separately for each class, provides a more comprehensive and reliable assessment of model performance when classes are imbalanced, as is frequently the case in biomedical scenarios.

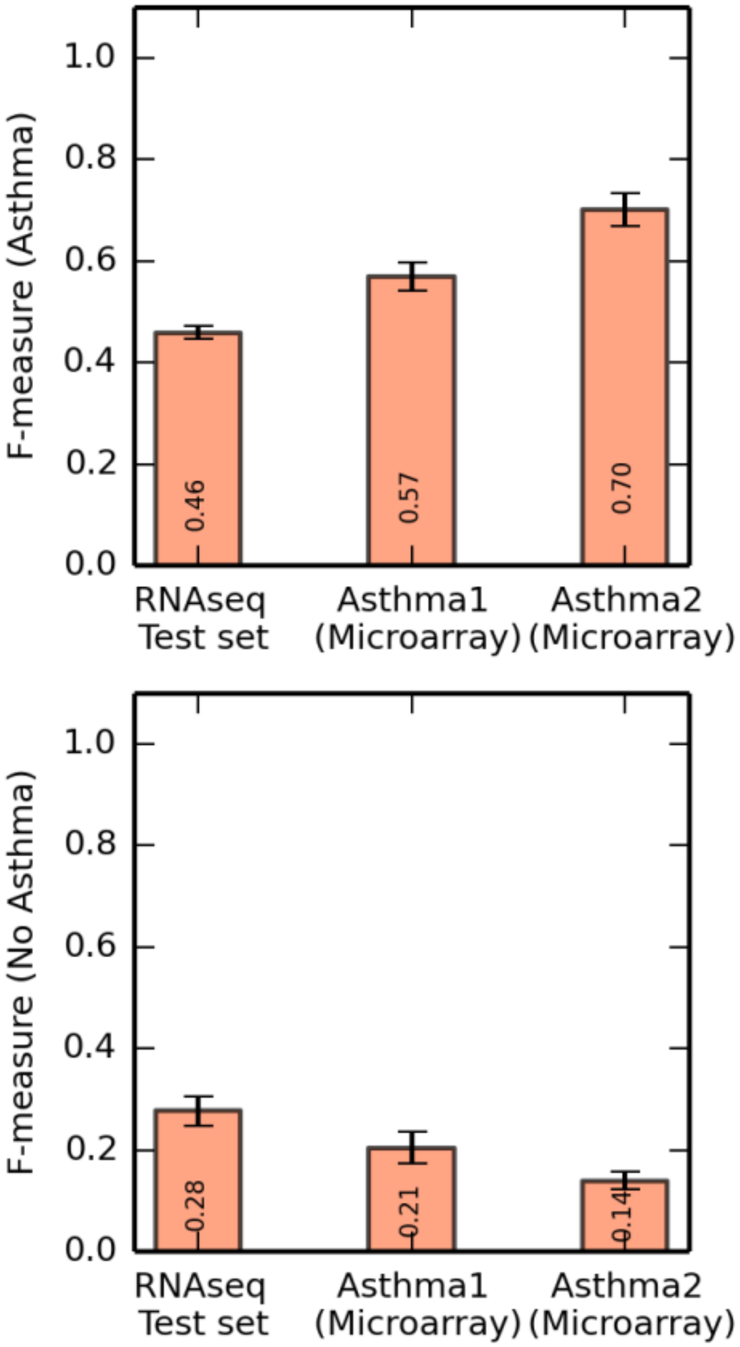

Supplementary Figure 6: Performance of permutation-based random classification models in test sets of independent subjects with asthma and controls. To determine the extent to which the performance of the classifier could have been due to chance, 100 permutation-based random models were obtained by randomly permuting the labels of the samples in the development set and executing each of the feature selection-global classification combinations on these randomized data sets in the same way as described above for the real development set. These random models were then applied to each of the asthma test sets considered in our study, and their performances were also evaluated in terms of the F-measure.

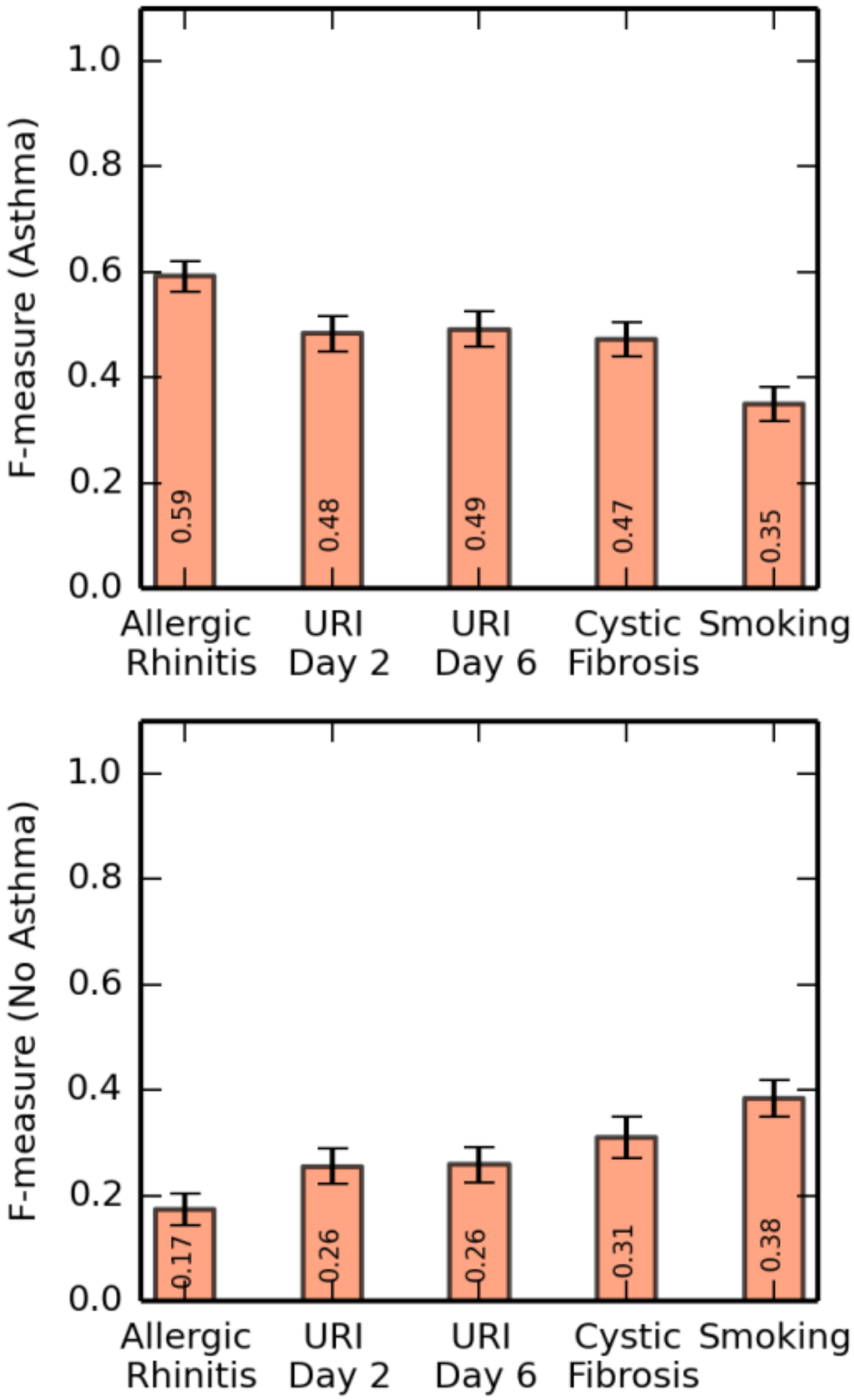

Supplementary Figure 7: Performance of permutation-based random classification models in test sets of independent subjects with non-asthma respiratory conditions and controls. 100 permutation-based random models were obtained by randomly permuting the labels of the samples in the development set and executing each of the feature selection-global classification combinations on these randomized data sets in the same way as described above for the real development set. These random models were then applied to these test sets, and their performances were also evaluated in terms of the F-measure.

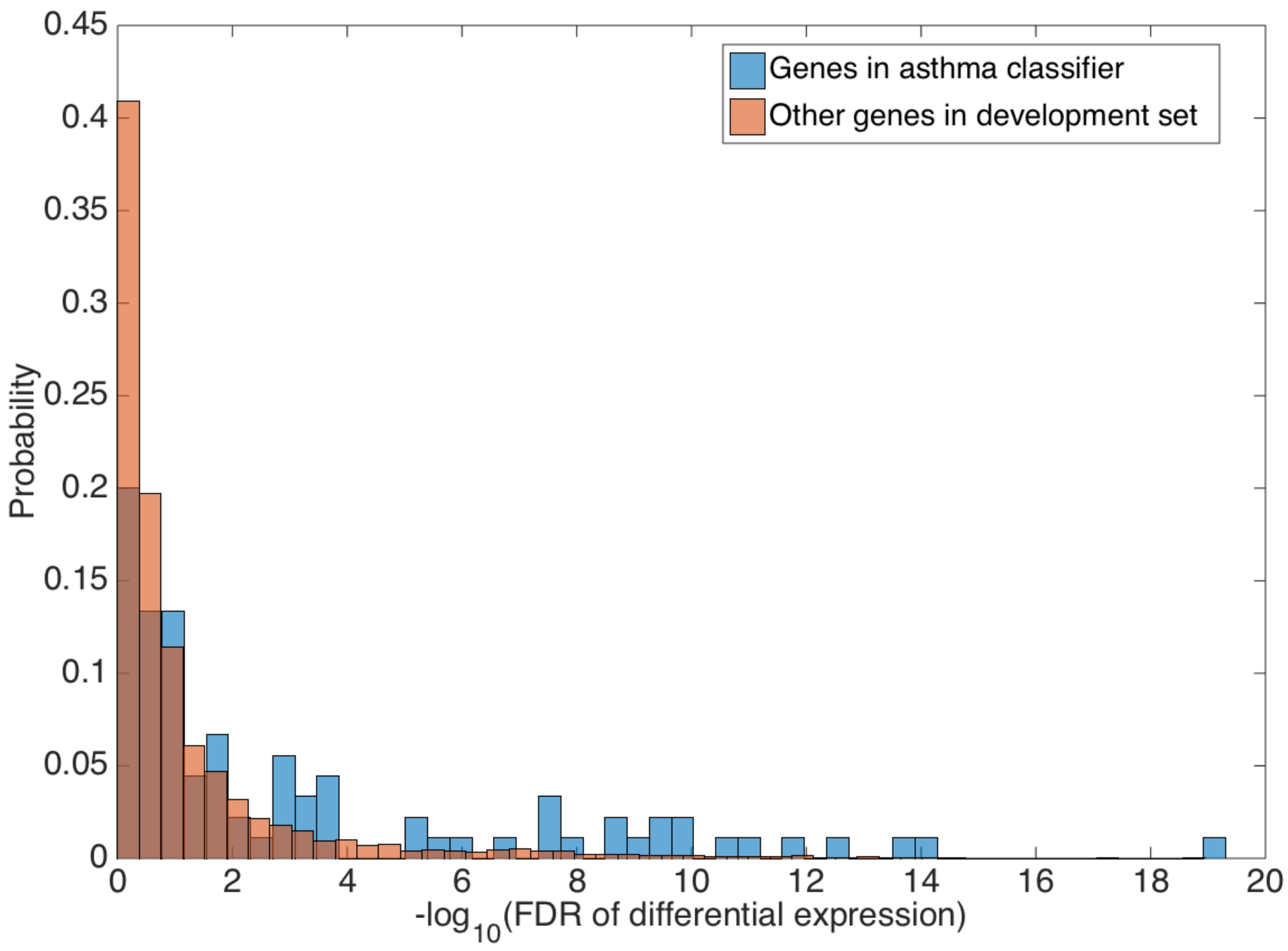

Supplementary Figure 8: Distribution of DESeq2 FDR values of differentially expressed genes in the asthma classifier (blue bars) vs. other genes in the RNAseq development set (coral bars). The Y-axis shows the probability of a gene having a –$\log_{10}$(FDR) value in the corresponding bin. This plot shows that the genes in the asthma classifier were likely to be more differentially expressed, i.e., higher -$\log_{10}$(FDR) or lower differential expression FDRs, than other genes in the development set.

Supplementary Table 3: Characteristics of the external asthma cohorts used for testing the asthma classifier

	**Asthma1[31]** GEO GSE19187			**Asthma2[32]** GEO GSE46171*		
Class	Asthma N = 13		No Asthma N=11	Asthma N=23		No Asthma N= 5
Definition	Recurring wheezing, dyspnea, cough and bronchodilator response		No personal or family history of atopy, rhinitis, or asthma	History of asthma		No known airway disease
Control	Controlled^	Uncontrolled	n/a	Controlled^	Uncontrolled	n/a
Subjects	7	6	11	16	7	5
Age - years	11.5 (3.2)	9.1 (0.6)	11.5 (3.1)	37 (19-66)†	29 (25-46) †	30 (18-37) †
Female	5 (71.4%)	2 (33.3%)	4 (36.4%)	36%	20%	14%
Race						
Caucasian	n/a	n/a	n/a	57%	82%	89%
African American	n/a	n/a	n/a	17%	9%	0%
Hispanic	n/a	n/a	n/a	13%	0%	0%
Asian/Other	n/a	n/a	n/a	13%	9%	11%
Rhinitis or atopic	7 (100%)	6 (100%)	0 (0%)	36%	16%	2%
FEV1 %predicted	97.6 (13.2)	78.2 (7.7)	n/a	97.8 (16.5)	91.2 (10.8)	98.3 (11.0)
FEV1/FVC	89.3 (5.6)	76.5 (3.2)	n/a	n/a	n/a	n/a
PC20 (mg/ml)	n/a	n/a	n/a	4.5 (5.1)	4.4 (5.2)	28 (27.1)

Results are number (%) or mean (SD) unless otherwise indicated

^For Asthma1, criteria for control per NAEPP/EPR3 criteria. For Asthma2, criteria for control not specified.

*For Asthma2, data that the authors deposited in GEO GSE46171 are a subset of their published results [32]. GSE46171 has data for 16 of the 23 subjects with controlled asthma, 7 of the 11 subjects with uncontrolled asthma, and 5 of the 9 controls reported in the authors' publication [32]. We indicate the number of subjects with publically available data (GSE46171) that were used in our analyses. The summary statistics shown are drawn from the authors' publication on their reported sample.

†Median (range)

Supplementary Table 4: Characteristics of the external cohorts with non-asthma respiratory conditions and controls used for testing the asthma classifier

	**Allergic Rhinitis**[35] GEO GSE43523*		**URI Day 2**[32] GEO GSE46171^		**URI Day 6**[32] GEO GSE46171^		**Cystic Fibrosis**[36] GEO GSE40445		**Smoking**[12] GEO GSE8987	
Class	Allergic Rhinitis N = 7	Control N=5	URI N=6	Control N=5	URI N= 6	Control N=5	Cystic Fibrosis N=5	Control N=5	Smoking N=7	Control N=8
Definition	Rhinitis symptoms and ≥1 elevated sIgE to aeroallergen	No symptoms, no sIgE to aeroallergen, total serum IgE < population mean	Day 2 following onset of "common cold" symptoms and no underlying airway disease	No URI symptoms and no known airway disease	Day 6 following onset of "common cold" symptoms and no underlying airway disease	No URI symptoms and no known airway disease	Homozygous F508del mutation	Overweight but healthy	≥10 cigarettes/day in past month and smoking ≥ 10 pack years	Never smoker, no environmental cigarette exposure and no respiratory symptoms
Age - years	37.9 (9.3)	32.9 (7.8)	30 (18-37)†	30(18-37)†	30 (18-37)†	30 (18-37)†	14 (4.2)	14.8 (1.1)	47 (12)	43 (18)
Female	60%	38.5%	14%	14%	14%	14%	3 (60%)	2 (40%)	1 (14.3%)	2 (25%)
Race										
Caucasian	0%	0%	89%	89%	89%	89%	5 (100%)	5 (100%)	3 (42.9%)	5 (62.5%)
Af- American	0%	0%	0%	0%	0%	0%	0%	0%	3 (42.9%)	2 (25%)
Hispanic	0%	0%	0%	0%	0%	0%	0%	0%	1 (14.3%)	1 (12.5%)
Asian/Other	100%	100%	11%	11%	11%	11%	0%	0%	0 (0%)	0 (0%)

Results are number (%) or mean (SD) unless otherwise indicated

*Data that the authors deposited in GEO GSE43523 are a subset of their published results [35]. GSE43523 has data for 7 of the 15 subjects with allergic rhinitis, and 5 of the 13 controls reported in the authors' publication [35]. We indicate the number of subjects with publically available data (GSE43523) that were used in our analyses. The summary statistics shown are drawn from the authors' publication on their reported cohort.

^Each subject provided a URI and control sample. The data that the authors deposited in GEO GSE46171 are a subset of their published results [32]. GSE46171 has data for 6 of the 9 healthy subjects reported in the authors' publication who provided samples during URI, and 5 of the 9 healthy subjects who provided samples after resolution of their URI [32]. We indicate the number of subjects with publically available data (GSE46171) that were used in our analyses. The summary statistics shown are drawn from the authors' publication on their reported cohort.

†Median (range)

Supplementary Table 5: **Basic functional annotations and references for asthma classifier genes that have been studied in the context of asthma and airway inflammation**

Gene	Annotation	References
ALOX15B	Member of lipoxygenase family whose members can affect bronchiolar constriction, cytokine secretion, and immune cell migration. The ALOX15B isoform may regulate cytokine secretion by macrophages and macrophage differentiation.	[1]
C3	Central role in classical and alternative complement pathway system activation; downstream effects include smooth muscle contraction, vascular permeability, histamine release.	[2]
CD177	Glycoprotein expressed by neutrophils. Used as a cell surface marker in studies of IL-17RB granulocytes in asthma.	[3, 4]
CDH26	One of eight genes targeted in a candidate gene study of asthma; negative results.	[5]
CDHR3	Variant in this gene associated with rhinovirus-induced wheezing and rhinovirus C illness. May function as a rhinovirus C receptor.	[6, 7]
CDKN1A	Mediator of microRNA-221-modulated airway smooth muscle hyperproliferation in cell culture studies of severe asthma.	[8]
CEBPD	CEBPD gene expression in bronchial specimens from asthma subjects associated with asthma susceptibility and inhaled corticosteroid treatment.	[9]
CLEC7A	Expression on CD11b+ dendritic cells plays a role in house dust mite-induced allergic airway inflammation in murine models.	[10]
CPA3	Mast cell mediator whose gene expression in epithelial brushings is upregulated in mild asthma and suppressed by corticosteroids in moderate asthma.	[11]
CYFIP2	One of 237 candidate genes targeted in a candidate gene study of asthma in Mexicans.	[12]
CYP1B1	One of 25 candidate genes targeted in a candidate gene study of xenobiotic-metabolizing enzymes in asthma among Russians.	[13]
DEFB1	Protein level elevated in induced sputum from severe asthmatics vs. controls. Also studied as one of 44 candidate genes in a candidate gene study of innate immune pathways in asthma and eczema among children from Boston and Connecticut.	[14, 15]
DUSP1	Expression in bronchial epithelial cell culture increased by dexamethasone, leading to suppression of p38 MAPK signaling and cytokine inhibition.	[16]
ESR1	SNPs in this gene associated with bronchial hyperresponsiveness and FEV1 decline, especially in females. miRNAs may impact pathogenesis of dust mite-induced asthma via regulation of ESR1.	[16-18]
FOS	Encodes a transcription factor involved in anti-inflammatory activity of steroid action in asthma.	[19, 20]
GSTT1	Modifies the impact of air pollution exposure on asthma.	[20]

IGF1	Increases airway inflammation, airway hyperresponsiveness, airway smooth muscle hyperplasia, and subepithelial fibrosis by interacting with ICAM-1, HIF-alpha activity, and VEGF expression.	[21]
LPHN1	SNP in LPHN1 associated with asthma and found to regulate airway smooth muscle cell adhesion and proliferation in vitro	[22]
LTBP1	siRNA knockdown of LTBP1 inhibited TGFbeta1 release in airway fibroblasts from asthma subjects.	[23]
MMP9	Released by neutrophils in allergic asthma subjects and in murine models of asthma.	[11, 24, 25]
NMU	Neuropeptide that amplifies Type 2 innate lymphoid cell-driven allergic lung inflammation in murine models.	[26]
S100A7	Antimicrobial peptide induced by IL-22 in T-cell lines derived from lung biopsy specimens of asthmatic subjects.	[27]
S100A8	Anti-apoptotic protein detected in supernatant of neutrophils treated with house dust mite extract and elevated in BAL from asthmatic vs. control subjects.	[28]
SCD	Inhibition of SCD in mice promoted airway hyperresponsiveness. SCD1 expression reduced in bronchial epithelial cells from asthma subjects vs. controls.	[29]
SCGB1A1	Levels in induced sputum higher in subjects with severe asthma vs. mild-moderate and healthy controls. BAL levels of SCGB1A1 correlated with epithelial detachment in bronchial biopsies.	[30]
SEMA5A	One of 11 genes mapped by 1000 of the top SNPs shared across European, African, and Hispanic populations in a rank-based analysis of shared genetic factors for asthma.	[31]
SERPINB3	*In vitro*-polarized Th2 cells from subjects with grass pollen allergy expressed higher mRNA levels of this serine protease inhibitor relative to CD27+CD4+ cells. Mediates mucus production in murine models of asthma.	[32, 33]
SERPINE2	Selected SNPs in this gene were associated with asthma and related traits.	[34, 35]
SLC26A4	Up-regulated in airway epithelial cells in association with mucus overproduction in murine models.	[36, 37]
SPRR1A	Intratracheal inoculation of mice with IL-13 induced more gene expression of SPRR1A than inhalation of IL-4.	[38]
TFPI	TFPI level concentration studied in 17 subjects with asthma during early and late stages of reaction.	[39]
TPSAB1	Mast cell biomarker used to subtype sputum subtypes in a study of eosinophilia and corticosteroid response in asthma.	[40]
TPSB2	Encodes mMCP-6, which is required for airway hyperresponsiveness in murine models of asthma.	[41]

Supplementary References

1. Mashima R, Okuyama T. The role of lipoxygenases in pathophysiology; new insights and future perspectives. Redox Biol 2015; 6:297-310.
2. Zhang X, Kohl J. A complex role for complement in allergic asthma. Expert Rev Clin Immunol 2010; 6:269-77.
3. Li L, Lukacs NW, Schaller MA, Petersen B, Baptist AP. IL-17RB(+) granulocytes are associated with airflow obstruction in asthma. Ann Allergy Asthma Immunol 2016; 117:674-9.
4. Ramirez-Velazquez C, Castillo EC, Guido-Bayardo L, Ortiz-Navarrete V. IL-17-producing peripheral blood CD177+ neutrophils increase in allergic asthmatic subjects. Allergy Asthma Clin Immunol 2013; 9:23.
5. Ferreira MA, Zhao ZZ, Thomsen SF, James M, Evans DM, Postmus PE, et al. Association and interaction analyses of eight genes under asthma linkage peaks. Allergy 2009; 64:1623-8.
6. Stenberg Hammar K, Niespodziana K, van Hage M, Kere J, Valenta R, Hedlin G, et al. Reduced CDHR3 expression in children wheezing with rhinovirus. Pediatr Allergy Immunol 2018; 29:200-6.
7. Bonnelykke K, Coleman AT, Evans MD, Thorsen J, Waage J, Vissing NH, et al. Cadherin-related Family Member 3 Genetics and Rhinovirus C Respiratory Illnesses. Am J Respir Crit Care Med 2018; 197:589-94.
8. Perry MM, Baker JE, Gibeon DS, Adcock IM, Chung KF. Airway smooth muscle hyperproliferation is regulated by microRNA-221 in severe asthma. Am J Respir Cell Mol Biol 2014; 50:7-17.
9. Sharma S, Kho AT, Chhabra D, Qiu W, Gaedigk R, Vyhlidal CA, et al. Glucocorticoid genes and the developmental origins of asthma susceptibility and treatment response. Am J Respir Cell Mol Biol 2015; 52:543-53.
10. Ito T, Hirose K, Norimoto A, Tamachi T, Yokota M, Saku A, et al. Dectin-1 Plays an Important Role in House Dust Mite-Induced Allergic Airway Inflammation through the Activation of CD11b+ Dendritic Cells. J Immunol 2017; 198:61-70.
11. Singhania A, Wallington JC, Smith CG, Horowitz D, Staples KJ, Howarth PH, et al. Multitissue Transcriptomics Delineates the Diversity of Airway T Cell Functions in Asthma. Am J Respir Cell Mol Biol 2018; 58:261-70.
12. Wu H, Romieu I, Shi M, Hancock DB, Li H, Sienra-Monge JJ, et al. Evaluation of candidate genes in a genome-wide association study of childhood asthma in Mexicans. J Allergy Clin Immunol 2010; 125:321-7 e13.
13. Polonikov AV, Ivanov VP, Solodilova MA. Genetic variation of genes for xenobiotic-metabolizing enzymes and risk of bronchial asthma: the importance of gene-gene and gene-environment interactions for disease susceptibility. J Hum Genet 2009; 54:440-9.
14. Sharma S, Poon A, Himes BE, Lasky-Su J, Sordillo JE, Belanger K, et al. Association of variants in innate immune genes with asthma and eczema. Pediatr Allergy Immunol 2012; 23:315-23.
15. Baines KJ, Wright TK, Simpson JL, McDonald VM, Wood LG, Parsons KS, et al. Airway beta-Defensin-1 Protein Is Elevated in COPD and Severe Asthma. Mediators Inflamm 2015; 2015:407271.

16. Keranen T, Moilanen E, Korhonen R. Suppression of cytokine production by glucocorticoids is mediated by MKP-1 in human lung epithelial cells. Inflamm Res 2017; 66:441-9.
17. Dong X, Xu M, Ren Z, Gu J, Lu M, Lu Q, et al. Regulation of CBL and ESR1 expression by microRNA-223p, 513a-5p and 625-5p may impact the pathogenesis of dust mite-induced pediatric asthma. Int J Mol Med 2016; 38:446-56.
18. Koppelman GH, Sayers I. Evidence of a genetic contribution to lung function decline in asthma. J Allergy Clin Immunol 2011; 128:479-84.
19. Patil RH, Naveen Kumar M, Kiran Kumar KM, Nagesh R, Kavya K, Babu RL, et al. Dexamethasone inhibits inflammatory response via down regulation of AP-1 transcription factor in human lung epithelial cells. Gene 2018; 645:85-94.
20. Liu S, Verma M, Michalec L, Liu W, Sripada A, Rollins D, et al. Steroid resistance of airway type 2 innate lymphoid cells from patients with severe asthma: The role of thymic stromal lymphopoietin. J Allergy Clin Immunol 2018; 141:257-68 e6.
21. Lee H, Kim SR, Oh Y, Cho SH, Schleimer RP, Lee YC. Targeting insulin-like growth factor-I and insulin-like growth factor-binding protein-3 signaling pathways. A novel therapeutic approach for asthma. Am J Respir Cell Mol Biol 2014; 50:667-77.
22. Faiz A, Donovan C, Nieuwenhuis MA, van den Berge M, Postma DS, Yao S, et al. Latrophilin receptors: novel bronchodilator targets in asthma. Thorax 2017; 72:74-82.
23. Zhou Y, Mirza S, Xu T, Tripathi P, Plunkett B, Myers A, et al. Aryl hydrocarbon receptor (AhR) modulates cockroach allergen-induced immune responses through active TGFbeta1 release. Mediators Inflamm 2014; 2014:591479.
24. Ventura I, Vega A, Chacon P, Chamorro C, Aroca R, Gomez E, et al. Neutrophils from allergic asthmatic patients produce and release metalloproteinase-9 upon direct exposure to allergens. Allergy 2014; 69:898-905.
25. Felsen CN, Savariar EN, Whitney M, Tsien RY. Detection and monitoring of localized matrix metalloproteinase upregulation in a murine model of asthma. Am J Physiol Lung Cell Mol Physiol 2014; 306:L764-74.
26. Wallrapp A, Riesenfeld SJ, Burkett PR, Abdulnour RE, Nyman J, Dionne D, et al. The neuropeptide NMU amplifies ILC2-driven allergic lung inflammation. Nature 2017; 549:351-6.
27. Pennino D, Bhavsar PK, Effner R, Avitabile S, Venn P, Quaranta M, et al. IL-22 suppresses IFN-gamma-mediated lung inflammation in asthmatic patients. J Allergy Clin Immunol 2013; 131:562-70.
28. Kim DH, Choi E, Lee JS, Lee NR, Baek SY, Gu A, et al. House Dust Mite Allergen Regulates Constitutive Apoptosis of Normal and Asthmatic Neutrophils via Toll-Like Receptor 4. PLoS One 2015; 10:e0125983.
29. Rodriguez-Perez N, Schiavi E, Frei R, Ferstl R, Wawrzyniak P, Smolinska S, et al. Altered fatty acid metabolism and reduced stearoyl-coenzyme a desaturase activity in asthma. Allergy 2017; 72:1744-52.
30. Emmanouil P, Loukides S, Kostikas K, Papatheodorou G, Papaporfyriou A, Hillas G, et al. Sputum and BAL Clara cell secretory protein and surfactant protein D levels in asthma. Allergy 2015; 70:711-4.

31. Ding L, Abebe T, Beyene J, Wilke RA, Goldberg A, Woo JG, et al. Rank-based genome-wide analysis reveals the association of ryanodine receptor-2 gene variants with childhood asthma among human populations. Hum Genomics 2013; 7:16.
32. Shamji MH, Temblay JN, Cheng W, Byrne SM, Macfarlane E, Switzer AR, et al. Antiapoptotic serine protease inhibitors contribute to survival of allergenic TH2 cells. J Allergy Clin Immunol 2017.
33. Sivaprasad U, Askew DJ, Ericksen MB, Gibson AM, Stier MT, Brandt EB, et al. A nonredundant role for mouse Serpinb3a in the induction of mucus production in asthma. J Allergy Clin Immunol 2011; 127:254-61, 61 e1-6.
34. Himes BE, Klanderman B, Ziniti J, Senter-Sylvia J, Soto-Quiros ME, Avila L, et al. Association of SERPINE2 with asthma. Chest 2011; 140:667-74.
35. Kerkhof M, Boezen HM, Granell R, Wijga AH, Brunekreef B, Smit HA, et al. Transient early wheeze and lung function in early childhood associated with chronic obstructive pulmonary disease genes. J Allergy Clin Immunol 2014; 133:68-76 e1-4.
36. Di Valentin E, Crahay C, Garbacki N, Hennuy B, Gueders M, Noel A, et al. New asthma biomarkers: lessons from murine models of acute and chronic asthma. Am J Physiol Lung Cell Mol Physiol 2009; 296:L185-97.
37. Nakao I, Kanaji S, Ohta S, Matsushita H, Arima K, Yuyama N, et al. Identification of pendrin as a common mediator for mucus production in bronchial asthma and chronic obstructive pulmonary disease. J Immunol 2008; 180:6262-9.
38. Finkelman FD, Yang M, Perkins C, Schleifer K, Sproles A, Santeliz J, et al. Suppressive effect of IL-4 on IL-13-induced genes in mouse lung. J Immunol 2005; 174:4630-8.
39. Kemona-Chetnik I, Bodzenta-Lukaszyk A, Kucharewicz I, Rogalewska AM. [Tissue factor and tissue factor pathway inhibitor during specific bronchial challenge in allergic asthma patients]. Przegl Lek 2005; 62:98-101.
40. Wang G, Baines KJ, Fu JJ, Wood LG, Simpson JL, McDonald VM, et al. Sputum mast cell subtypes relate to eosinophilia and corticosteroid response in asthma. Eur Respir J 2016; 47:1123-33.
41. Cui Y, Dahlin JS, Feinstein R, Bankova LG, Xing W, Shin K, et al. Mouse mast cell protease-6 and MHC are involved in the development of experimental asthma. J Immunol 2014; 193:4783-9.
